# Novel *SPEG* variants in a neonate with severe dilated cardiomyopathy and relatively mild hypotonia

**DOI:** 10.1038/s41439-023-00253-w

**Published:** 2023-09-06

**Authors:** Hana Milena Fujimoto, Masanori Fujimoto, Takahiro Sugiura, Shigeharu Nakane, Yasuhiro Wakano, Emi Sato, Hironori Oshita, Yasuko Togawa, Mari Sugimoto, Takenori Kato, Kazushi Yasuda, Kanji Muramatsu, Shinji Saitoh

**Affiliations:** 1 Department of Pediatrics, Toyohashi Municipal Hospital, Toyohashi, Aichi Japan; 2 Department of Pediatrics and Neonatology, Nagoya City University Graduate School of Medical Sciences, Nagoya, Japan; 3 Department of Cardiology, Aichi Children’s Health and Medical Center, Obu, Aichi Japan

**Keywords:** Neuromuscular disease, Genetic testing

## Abstract

*Striated muscle preferentially expressed protein kinase* (*SPEG*) variants have been reported to cause centronuclear myopathy associated with cardiac diseases. The severity of skeletal muscle symptoms and cardiac symptoms are presumably related to the location of the variant. Here, we report novel *SPEG* compound heterozygous pathological variants in a neonate with severe dilated cardiomyopathy and relatively mild hypotonia. This report expands the genotype-phenotype correlations of patients with *SPEG* variants.

Centronuclear myopathy (CNM) is a form of congenital myopathy characterized by muscle weakness and centralized skeletal muscle nuclei on biopsy^1^. The clinical presentation of patients ranges from severe muscle weakness requiring respiratory support to mild hypotonia. Recessive variants in *striated muscle preferentially expressed protein kinase* (*SPEG*), a member of the myosin light chain kinase family, have recently been reported to cause a CNM phenotype with or without cardiac complications, such as dilated cardiomyopathy (DCM)^2–4^. It is known that some patients have more severe cardiac symptoms than skeletal muscle symptoms, but what determines their phenotype remains unclear. We herein report novel *SPEG* compound heterozygous pathological variants in a neonate with severe DCM and relatively mild hypotonia. This report expands the genotype-phenotype correlations of patients with *SPEG* variants.

The patient was a boy born at 36 weeks and 6 days of gestation with a birth weight of 2668 g (45.1 percentile) who was the second child of nonconsanguineous parents. There was no family history of neuromuscular disease (Fig. 1A). During pregnancy, antenatal ultrasound examination revealed pleural effusion and ascites, and he was suspected to have left ventricular noncompaction. After he was born by spontaneous vaginal delivery, he was admitted to the neonatal intensive care unit for close examination. On admission, he showed severe hypotonia (Fig. 1B), and echocardiography detected abnormal trabeculation of the left ventricle with marked thickening of the noncompacted layer, which was consistent with left ventricular noncompaction. The amount of pericardial effusion was small, and his cardiac function was preserved. The amount of pleural effusion increased gradually, and drainage revealed chylothorax. After administration of medium-chain fatty acid milk, the effusion decreased. The patient was temporarily on tube feeding due to hypotonia, but his muscle tone improved by approximately two weeks after birth, and he was gradually able to feed orally. However, his cardiac function deteriorated three weeks after birth, and dilation of the left ventricle became apparent (Fig. 1C, D). His cardiac function was not improved by intravenous inotropic agents or diuretics, and we switched to chronic heart failure management with beta blockers, angiotensin II receptor blockers and phosphodiesterase inhibitors. Myocardial biopsy performed during cardiac catheterization revealed fibrosis of the interstitium associated with dropout of cardiomyocytes (Fig. 1E). Despite careful adjustment of medications, he died at five months of age due to heart failure.

**Fig. 1:**
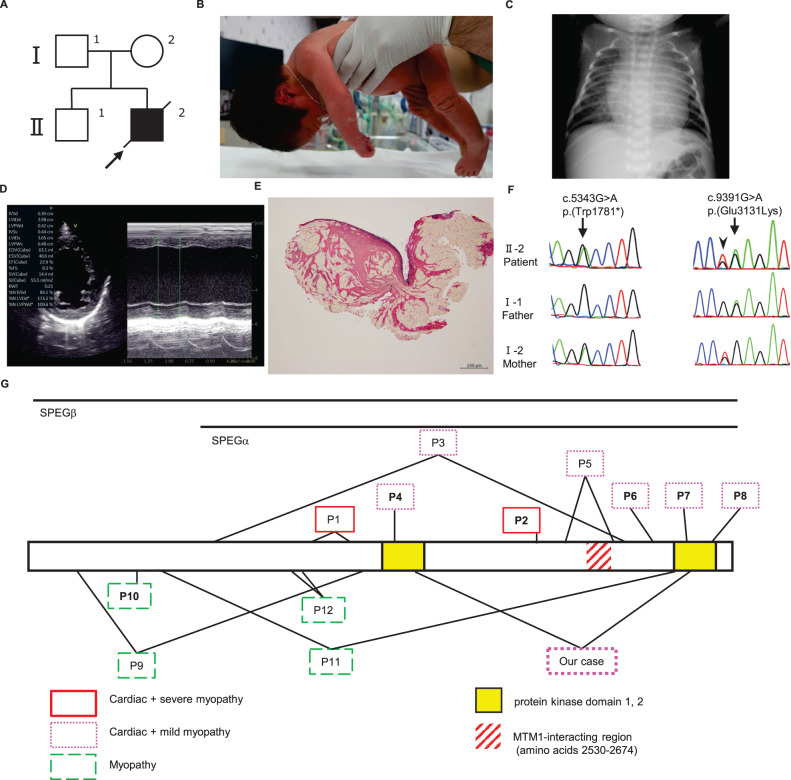
Clinical presentation of our case and reported SPEG variants. **A** The family pedigree. The arrow shows the patient reported. **B** A photograph of the patient taken at one week of age. At this time, he was floppy and needed tube feeding. X-ray (**C**) and echocardiography (**D**) of the patient when heart failure was detected showing cardiac enlargement, ventricular dilation and decreased cardiac contractility. **E** Elastica‐Picrosirius red staining of the myocardium. The red stained area shows fibrosis of the interstitium associated with myocardial dropout. **F** Sanger sequencing showing the variants detected, revealing that c.5343 G > A (p.(Trp1781*)) was inherited from the mother and that c.9391 G > A (p.(Glu3131Lys)) was inherited from the father. The variant marked with an arrowhead next to c.9391 G > A (arrow) is a single-nucleotide polymorphism derived from his mother that has no pathological significance. **G** A schema of the full-length striated muscle preferentially expressed protein kinase (SPEG) protein showing the protein kinase domain and MTM1-interacting region. The variants reported thus far are indicated (homozygous variants in bold). Variants causing severe symptoms of both the heart and skeletal muscle (P1-2) are shown in red boxes with solid lines. Those with severe cardiac symptoms and milder or no skeletal muscle symptoms (P3-8 and our case) are shown in pink boxes with dotted lines. Those with skeletal muscle symptoms but with milder or no cardiac symptoms (P9-12) are shown in green boxes with dashed lines.

A chromosome test was performed to search for underlying disease, which revealed a normal male karyotype (46,XY). Newborn screening for metabolic disorders was negative. Next, we performed whole-exome sequencing. After obtaining written informed consent from his parents, we collected blood samples from the patient. Genomic DNA was extracted from peripheral blood samples following a standard protocol, and exome sequencing was performed for the patient using an Ion AmpliSeq Exome RDY kit (Thermo Fisher Scientific) according to the manufacturer’s protocols. The results showed a nonsense variant [NM_005876.5: c.5343 G > A: p.(Trp1781*)] and a missense variant [NM_005876.5: c.9391 G > A: p.(Glu3131Lys)] in *SPEG*, neither of which have been previously reported in Genome Aggregation Database (gnomAD, URL: http://gnomad.broadinstitute.org/). Standard Sanger sequencing was performed for the patient and parents, revealing that p.(Trp1781*) and p.(Glu3131Lys) were inherited from the mother and the father, respectively (Fig. 1F). The patient’s phenotype matched the known spectrum of clinical features for *SPEG* variants. The maternally derived nonsense variant is predicted to be pathogenic according to American College of Medical Genetics and Genomics and the Association for Molecular Pathology guidelines (PVS1: nonsense, PM2: absent from controls, PP4: patient’s phenotype)^5^. The paternally derived missense variant is predicted to be damaging by in silico evaluation tools, Combined Annotation Dependent Depletion (CADD score 31) and PolyPhen-2 (score 1.000) and is interpreted as likely pathogenic according to guidelines (PM2: absent from controls, PM3: in *trans* with a pathogenic variant, PP3: in silico evaluation, PP4: patient’s phenotype)^5^.

SPEG plays a significant role in muscle development and regeneration. In addition, it regulates cardiac and skeletal muscle calcium homeostasis and is involved in both acquired and genetic cardiovascular disease^3^. SPEG has two protein kinase domains, 1 and 2, which are reported to be key domains that control Ca^2+^ reuptake^6,7^. To date, 15 pathogenic variants of *SPEG* have been reported^3,4,8–13^. The 12 variants that were evaluated and monitored for both neuromuscular manifestations and cardiac abnormalities are shown in Fig. 1G and Table 1, along with data from our patient. We defined “severe myopathy” as a group of patients with severe muscle weakness requiring respiratory support and “mild or no myopathy” as a group of patients with a milder phenotype. Because we did not conduct skeletal muscle biopsy or postmortem pathology autopsy, we presumed that the patient had CNM due to the presence of hypotonia.

**Table 1:** Reported *SPEG* variants categorized according to phenotype.

			SPEGα	MTM1
Cardiac + severe myopathy				
P1	p.(Thr1237Serfs*46)	p.(Arg1426*)	−/−	−/−
P2	p.(Tyr2373*)		−/−	−/−
Cardiac + mild or no myopathy				
P3	p.(Ala972Aspfs*79)	p.(Gly2757Val)	−/−	−/+
P4	**p.(Glu1680Lys)**		−/−	+/+
P5	p.(Arg2470Ser)	p.(Pro2687Thr)	−/−	+/+
P6	p.(Arg2958*)		−/−	?/?
P7	**p.(Val3062del)**		−/−	+/+
P8	p.(Arg3196*)		−/−	+/+
Our case	p.(Trp1781*)	p.(Glu3131Lys)	−/−	−/+
Myopathy				
P9	p.(Lys359Valfs*35)	p.(Arg1467*)	+/−	+/−
P10	p.(Thr544Aspfs*48)		+/+	+/+
P11	p.(Leu728Argfs*82)	p.(Val2997Glyfs*52)	+/−	+/+
P12	p.(Asp1196fs)	c.3715+4 C>T	−/?	−/?

Homozygous variants shown in bold. *SPEG*: striated muscle preferentially expressed protein kinase; SPEGα (+): at least one allele is assumed to have normal SPEGα; SPEGα (–): both SPEGα alleles are assumed to be affected; MTM1 (+): at least one SPEGα is assumed to have a normal MTM1-interacting region; MTM1 (–): there is no unaffected MTM1-interacting region; ? uncertain.

It has been hypothesized that the phenotype of patients with *SPEG* variants may be predicted by the location of the variants. This is explained by two known theories. First, the severity of cardiac comorbidities has been suggested to be linked to variants affecting both isoforms expressed in skeletal and cardiac muscle^4,12^. Second, a variant that involves a certain region in *SPEG* called the MTM1-interacting region is assumed to contribute to the severity of skeletal muscle symptoms^2,8^.

Two of the four isoforms of *SPEG*, *SPEGα* and *SPEGβ*, are expressed in skeletal and cardiac muscle^14^ and play a critical role in regulating their contraction by phosphorylating Ca^2+^-handling proteins and maintaining the transverse tubule^3^. The severity of cardiac comorbidities is suggested to be linked to variants affecting both *SPEGα* and *β*^4,12^. If the variant in at least one allele is in a position involving only *SPEGβ*, cardiac function tends to be mostly preserved because it is complemented by *SPEGα* (Fig. 1G, Table 1). Consistent with this theory, we observed that Patients (P) 9-12 had preserved cardiac function but that P1-8 exhibited severe cardiac dysfunction, such as DCM. In our case, the severe cardiac manifestation might be explained by the effects of the variants on both the *α* and *β* isoforms.

On the other hand, some patients have milder skeletal dysfunction compared to their severe cardiac dysfunction. This is presumably caused by the MTM1-interacting region of *SPEG*^2,8^. *SPEG* variants were first reported to cause the CNM phenotype in the process of investigating MTM1 function and related proteins^2^. In striated muscle, the protein encoded by *MTM1*, a causative gene of X-linked myotubular myopathy, interacts and colocalizes with a particular region in SPEG (NM_005876.5:c.2530-2674). This MTM1-interacting region in *SPEG* plays a critical role in excitation-contraction coupling and cytoskeletal organization^2^. For patients who have variants affecting both *SPEGα* and *β* isoforms, it has been speculated that patients, such as P1-P2 with truncating mutations N-terminal to the MTM1-interacting region tend to have severe myopathic symptoms, such as the need for respiratory support. In contrast, patients, such as P3-P8, who have one or both alleles with an intact MTM1-interacting region present with relatively mild or no skeletal muscle symptoms, suggesting that missense variants or deletions are unlikely to cause a change in the 3D structure (Fig. 1G, Table 1)^2,8,13^. In our case, the MTM1-interacting region of the allele of the missense variant p.(Glu3131Lys) should be intact because the variant is located outside of the MTM1-interacting region. The other variant in our case, p.(Trp1781*), is located N-terminal to the MTM1-interacting region, and a truncated protein should not contain the MTM1-interacting region and might be affected by nonsense-mediated mRNA decay. Relatively mild myopathic symptoms in a patient may be explained by the presence of the MTM1-interacting region.

In conclusion, novel compound heterozygous *SPEG* variants were identified in an infant developing dilated cardiomyopathy who had transient hypotonia. The variants and phenotype in this case support and reinforce the hypothesis that has been postulated in previous reports and expand the genotype-phenotype correlations of patients with *SPEG* variants.

## Data Availability

The relevant data from this Data Report are hosted at the Human Genome Variation Database at 10.6084/m9.figshare.hgv.3324. 10.6084/m9.figshare.hgv.3327.

## References

[1] Jungbluth H, Wallgren-Pettersson C, Laporte J (2008). Centronuclear (myotubular) myopathy. Orphanet J. Rare Dis..

[2] Agrawal P (2014). SPEG interacts with myotubularin, and its deficiency causes centronuclear myopathy with dilated cardiomyopathy. Am. J. Hum. Genet.

[3] Campbell H, Aguilar-Sanchez Y, Quick A, Dobrev D, Wehrens X (2021). SPEG: a key regulator of cardiac calcium homeostasis. Cardiovasc Res.

[4] Luo S, Rosen S, Li Q, Agrawal P (2021). Striated preferentially expressed protein kinase (SPEG) in muscle development, function, and disease. Int J. Mol. Sci..

[5] Richards S (2015). Standards and guidelines for the interpretation of sequence variants: a joint consensus recommendation of the American College of Medical Genetics and Genomics and the Association for Molecular Pathology. Genet Med.

[6] Quick A (2017). ‘Striated muscle preferentially expressed protein kinase’ (SPEG) is essential for cardiac function by regulating junctional membrane complex activity. Circ. Res.

[7] Quan C (2019). SPEG controls calcium reuptake into the sarcoplasmic reticulum through regulating SERCA2a by its second kinase-domain. Circ. Res.

[8] Gurgel-Giannetti J (2021). A novel SPEG mutation causing congenital myopathy with fiber size disproportion and dilated cardiomyopathy with heart transplantation. Neuromuscul. Disord..

[9] Levitas A (2020). A novel mutation in SPEG causes early onset dilated cardiomyopathy. PLoS Genet.

[10] Zhang G (2021). Clinical and genetic analysis of a case with centronuclear myopathy caused by SPEG gene mutation: a case report and literature review. BMC Pediatr..

[11] Jaouadi H (2022). Dilated-left ventricular non-compaction cardiomyopathy in a pediatric case with SPEG compound heterozygous variants. Int J. Mol. Sci..

[12] Qualls A (2019). Novel SPEG mutations in congenital myopathies: genotype-phenotype correlations. Muscle Nerve.

[13] Wang H (2017). Insights from genotype-phenotype correlations by novel SPEG mutations causing centronuclear myopathy. Neuromuscul. Disord..

[14] Hsieh C (2000). Striated muscle preferentially expressed genes alpha and beta are two serine/threonine protein kinases derived from the same gene as the aortic preferentially expressed gene-1. J. Biol. Chem..

